# Supplementary material for: Are carbon emissions trading and green financial instruments synergistic? -Comprehensive quantitative research based on content analysis
Source: PLoS One. 2024 Mar 7;19(3):e0298601. doi: 10.1371/journal.pone.0298601 (PMC10919601; doi:10.1371/journal.pone.0298601)
Supplement: S1 Data — (ZIP) [file pone.0298601.s001.zip › Data/地级市Z_F工作报告.zip!/╦─┤¿/╡┬╤⌠╩╨/└┤╫╘╣1⁄2╓┌║┼í╛┬φ┐╦╩2╛▌═°í┐_2016.docx]

各位代表：

我代表市人民政府，向大会报告工作，请各位代表审查，并请各位政协委员提出意见。

2016年工作回顾

2016年是不平凡的一年。面对错综复杂的宏观形势和繁重艰巨的改革任务，在省委、省政府和市委的坚强领导下，在市人大、市政协的监督支持下，我们坚持稳中求进工作总基调，主动适应经济发展新常态，牢牢把握重大战略机遇，积极应对各种困难挑战，经济社会发展稳中有进、稳中提质，实现了“十三五”良好开局。接受国务院第三次大督查受到高度评价，转型升级相关典型经验被国务院通报表扬。全市实现地区生产总值1752.5亿元、增长8.4%，总量与增速均居全省第三。一般公共预算收入突破百亿大关、增长12.9%，社会消费品零售总额698.8亿元、增长13.5%，城乡居民人均可支配收入分别增长7.8%、9.1%，节能减排完成省定目标。

一、经济呈中高速增长，产业迈向中高端水平

“项目年”工作成效显著。全社会固定资产投资跨越千亿级，达到1125.1亿元，增长14.7%，增速居全省第三。着力谋项目，纳入“十三五”规划重大项目2211个，总投资7823亿元；争取国家专项建设基金27.87亿元，增长3倍；争取国省预算内资金6.87亿元，增长20%。着力引项目，到位市外资金700亿元，新引进5亿元及以上重大项目55个；推出PPP项目5个，吸引社会投资312亿元，录入财政部PPP综合信息平台的项目投资额居全省第一。着力建项目，100个省市重点项目全部完成年度投资目标，四川城市轨道交通学院等一批项目开工建设，成都经济区环线高速德阳段等一批项目加快推进，明日宇航数控中心、万达广场等一批项目竣工投用。

产业转型升级步伐加快。规上工业增加值增长9.7%，主营业务收入突破3000亿元。改造提升传统产业，实施重大产业转型升级项目60个，完成技改投资366.2亿元、增长9.5%；医药、机械、建材等产业保持两位数增速。战略性新兴产业强势上扬，增长15.1%、提高11.8个百分点。服务业发展势头向好，实现增加值556.5亿元、增长9%，新建现代金融产业园，建成跨境电商产业孵化中心，“中欧班列”德阳现代物流港加快推进。实现旅游收入190.11亿元、增长22.1%。金融机构各项存款余额2327.7亿元、增长11.7%，各项贷款余额1198.7亿元、增长9.4%。现代农业发展加快，建成万亩农机化生产技术核心示范区8个、高标准农田7.46万亩，开展全域永久基本农田划定工作，粮食实现“十三连增”。

全创改革迈出坚实步伐。高新技术产业总产值达1132.5亿元，居全省第三。狠抓全面创新改革“一号工程”，出台领军人才“1+3”政策，设立专项资金4亿元，建成全面创新（国际）领军人才服务中心，引进领军人才15名，入选国家智能制造应用示范项目2个，军民融合实现产值1300亿元、增长11%。创新能力不断提升，新增国家重点实验室1个、国家级企业技术中心1个，新增专利授权2205件、增长28%，获批全国首批专利质押融资及专利保险试点城市，成立高端装备制造行业协会、石墨烯产业创新联盟，建立铝空动力产业园，“德阳装备制造工业云”入驻企业1400余家。创新创业蓬勃开展，新增国家级众创空间2个，建成国家级海峡两岸青年创业基地，开展“德阳创客”主题活动9场，新增创新型企业85家。

二、城乡建设提档升级，全域城镇化开启新篇

成德同城化纵深推进。狠抓“六个协同”，同城化全面提速。《成德同城化空间发展战略规划》形成初步成果。“五环多轴”交通体系建设全面启动，天府大道北延线、什德中、德罗、德绵干道等一批重点项目开工，成德动车实现公交化运营。全域推进“城市升级三年行动”，完成长江路、庐山路、凯江路等示范段改造，沱江路下穿城际铁路和宝成铁路、旌南湿地公园等一批项目全面完成，城市面貌焕然一新。与成都合作组建农村产权交易所，成德工业园聚集规上企业40家。

全域城镇化取得新进展。全市城镇化率达49.6%，新增建成区面积4平方公里。城市总体规划修编有序推进，完成中心城区综合管廊、污水再生利用等专项规划，启动海绵城市等专项规划。调整完善土地利用总规，城乡建设用地增减挂钩试点深入推进。智慧城市建设步伐加快，政务云、智慧医疗、智慧社区等一批惠民项目投入使用。卫星县城承载力进一步增强，“多规合一”试点加快推进。罗江撤县设区进入国家审批阶段。中江县成为全省首次命名的5个县域经济发展模范县之一。“百镇建设行动”扎实推进，完成投资3.2亿元，就地吸纳农业转移人口1.47万人。新建幸福美丽新村200个。

生态文明建设切实加强。出台生态文明体制改革方案，加快建设美丽德阳。集中力量打好污染防治“三大战役”，完成水污染物综合整治项目188个，国控省控断面水质明显改善；采取一切可行措施，切实加强大气污染防治；实施土壤污染物修复防控项目34个，综合治理面积2.2万亩。什邡建立全省首支县级环保警察中队。节能减排财政政策综合示范市建设扎实推进，实施典型示范项目126个。德阳经开区被确定为国家循环化改造示范试点园区。启动大规模绿化行动，全市林木覆盖率40.83%。成功申办第八届中国月季展。全国文明城市创建工作深入推进。

三、深化改革蹄疾步稳，开放水平进一步提升

供给侧改革扎实推进。出台18条措施和“1+5”方案，“三去一降一补”成效明显。去产能有序推进，淘汰落后产能企业44户，退出炼钢产能160万吨、煤炭产能60万吨，超额完成省定目标。去库存进展顺利，出台鼓励住房消费政策，商品住房销售面积增长22.8%，去化周期降至合理水平。去杠杆力度加大，市内企业实现直接融资360亿元，新增新三板挂牌企业8家、成都（川藏）股权交易中心挂牌企业175家，发放应急转贷资金192笔31亿元。降成本措施有力，落实企业税收优惠政策、减负34亿元，落实直购电直供气优惠政策、减负2亿元，降低社会保险费率、减负5.3亿元，兑现失业保险稳岗补贴8400余万元。补短板扎实推进，完成农村交通、水利、电网等基础设施投资12亿元。

重点领域改革迈向深入。新增国家级改革试点7个、省级改革试点3个。推进国资国企改革，停产企业“人资分离”改革进入实质操作阶段，市建投、市产投集团挂牌运营。助力国企轻装上阵，中国二重实现扭亏脱困。深化“放管服”改革，探索相对集中行政许可权改革，全面取消非行政许可审批事项，在全省率先实施“五证合一”，启动综合行政执法体制改革试点，全面推行“双随机、一公开”，创新实施“一诺即办”容缺预审，新登记市场主体2.08万户，民营经济比重超60%。建成市县一体化公共资源交易平台。不动产统一登记工作有序推进。农村土地“三权分置”改革扎实开展，流转土地63万亩、占全市耕地面积25%。

内陆开放高地建设加快。全市进出口总额17.6亿美元。服务贸易快速发展，实绩企业超过150家，实现服务贸易额1.8亿美元，居全省第三。成功举办中国制造2025对话德国工业4.0大会、医药产业与健康城市高峰论坛、德阳（香港）投资推介会等活动，积极参加第十六届西博会、“万企出国门”“千企行丝路”等活动，签订中国微创国际医学中心、中国非物质文化遗产传承中心、德国欧绿保零能耗国家级静脉产业园等一批重大项目，中德应急产业园、中捷中小企业产业园等国别合作园区加快推进。外事、台侨、海关、检验检疫等工作得到加强。第二轮援藏暨援彝工作正式启动。

四、全面小康扎实推进，民生福祉全方位改善

民生保障稳步提高。全面完成民生工程、民生实事，实现民生支出152.4亿元，占一般公共预算支出比重达67%。脱贫攻坚成效明显，减贫4.5万人，市级贫困村退出100个，职教“三免三定”惠及贫困学生500余名。城镇新增就业4.2万人，城镇登记失业率3.9%。全国首批全民参保试点工作圆满收官，各类社会保险参保率达89%，城乡居民基本医疗保险制度顺利实现整合，退休职工基本养老金待遇进一步提高，城乡居民低保标准分别增长15.5%、24%。改善义务教育薄弱学校，新建校舍3.11万平方米，发放各类助学资金3.5亿元。新增(改造)养老床位3550张，建成日间照料中心103个。开工保障性安居工程9953套、竣工10090套，棚改货币化安置率近60%。

社会事业全面发展。新建普惠性民办幼儿园9所。县域内义务教育均衡发展，什邡通过国家督导认定，中江通过省级督导评估。高考本科上线突破万人、上线率保持全省第三。西南财大天府学院加快建设，四川司法警官学院启动迁建。开展国家医养结合试点和健康城市建设评价试点，启动国家城市公立医院综合改革试点，重大传染病疫情有效防控，人口自然增长率1.8‰。全民健身、竞技体育取得新进步，德阳女子拳击队任灿灿获里约奥运会女子拳击铜牌，创历史之最。公共文化服务体系进一步健全，成功举办三星堆戏剧季、全国散文名家走进德阳等活动百余场。“德善之城”建设扎实推进，网络众筹项目6个。荣获全国残疾人工作先进单位称号。

社会治理水平不断提升。以“主动警务战略”为核心的平安德阳建设深入推进，社会大局保持和谐稳定。科学制定“七五”普法规划，“法律七进”扎实推进，获评全国法治宣传教育先进城市。妥善处置信访积案，健全“大调解”工作体系，化解纠纷2.7万余件。基层治理迈出新步伐，创建省级“村民自治模范单位暨和谐社区建设示范单位”11个。安全生产事故持续下降，食品和农产品“双安双创”工作走在全省前列，防灾减灾和应急管理体系进一步完善。持续深化“双拥在基层”活动，成功创建“全国双拥模范城”。

与此同时，我们扎实开展“两学一做”学习教育，进一步转变政府职能，持续改进工作作风，政府执行力明显增强。自觉接受市人大及其常委会法律监督、工作监督和市政协的民主监督，以及社会各界监督，办理人大代表建议121件、政协提案252件。法治政府建设深入推进，规范行政执法行为等工作进一步加强。保持正风反腐高压态势，落实“一诺三清”廉政制度，查处违纪违法案件765件，“三公”经费压减20%以上。

统计调查、审计、住房公积金、档案、民族宗教、妇女儿童、青年、老龄、人防、保密、地方志、气象、红十字会等工作取得新进展。

过去五年发展回顾

各位代表，2016年是本届政府任期的最后一年。回顾五年的历程，我们倍感欣慰！全市上下风雨同舟、砥砺奋进，以更加坚实的步伐、令人瞩目的业绩，成就了一个辉煌的五年。尤为可喜的是，德阳成为国家全面创新改革试验区，成德同城化纳入成都平原经济区“十三五”规划，为推进我市在高起点上新发展注入了强劲动力。

五年来，我们致力于稳增长、调结构，经济发展迈入“双中高”新阶段。综合实力显著增强，地区生产总值年均增长9.7%、增速高于全国全省，固定资产投资累计突破4500亿元、年均增长11.6%，一般公共预算收入年均增长8.3%。产业结构持续优化，规上工业、服务业增加值年均分别增长11.5%、9.8%；服务业增加值占GDP比重达31.8%，比2011年提高7.4个百分点；社会消费品零售总额是2011年的近2倍；人均粮食占有量、粮食单产等指标居全省第一。经济新动能加快成长，战略性新兴产业占工业比重比2011年提高1.5个百分点；上市挂牌企业达270家，比2011年增加265家。

五年来，我们致力于抓创新、促转型，创新驱动迈入换档提质新阶段。成功跻身国家节能减排财政政策综合示范市，成功创建国家知识产权示范市，荣膺中国科协创新驱动示范市、全国首个职业教育促进经济社会发展示范区。创新驱动多点发力，德阳高新区升格为国家级高新区，高新技术产业总产值突破千亿大关、居全省第三，科技进步对经济增长的贡献率超过50%，全社会研发投入占GDP比重达2.5%、居全省第二，企业技术中心数量实现翻番，全国新增的14座百万千瓦级核电站有12座由德阳制造。创新创业掀起热潮，五年累计新登记市场主体6.9万户、年均增长18.72%，各类创投资金规模超50亿元，“德阳创客”成为城市新名片。

五年来，我们致力于打基础、利长远，城乡建设迈入跨越发展新阶段。基础设施投资累计突破千亿元，年均增长16.3%，城镇化率接近50%、比2011年提升6.6个百分点，建成区面积净增63.67平方公里。基础设施明显改善，城际铁路建成通车，新增高速公路、干线公路8条，新（改）建农村公路1690公里，新增普通国省道12条666公里、增幅达195%；开工建设中型水库3座，建成各类水利工程14056处。城乡环境更加优美，成功创建国家卫生城市、园林城市、森林城市和四川省文明城市，列入全国重点镇10个、特色景观旅游名镇2个，建成幸福美丽新村718个，林木覆盖率比2011年提高1.83个百分点。

五年来，我们致力于促改革、增活力，改革开放迈入全域推进新阶段。累计启动272项改革事项，59项省级以上改革试点扎实推进，农村集体资产产权制度改革、中国二重司法重整、职业教育综合改革、“一窗进出”审批模式等受到中央和省委充分肯定。开放合作全方位推进，累计引进市外到位资金3225.3亿元、利用外资9.27亿美元，境外投资企业达55家，参与国际产能合作企业160余家，德阳海关、出入境检验检疫局开关开检，创建国家级出口基地4个，进出口额累计超过150亿美元、居全省第二，国际友好城市达7个，发展活力进一步显现。

五年来，我们致力于办实事、惠民生，人民生活迈入全面改善新阶段。累计民生支出658.3亿元、年均增长12%，减贫37.05万人。城乡居民人均可支配收入分别增长1.5倍、1.8倍。基本实现人人享有社会保险，城乡低保标准分别提高94%、176%。建成保障性安居工程87685套，改造农村危房2.3万户，解决农村安全饮水45.9万人。累计办理人大代表建议555件、政协提案1255件，城镇居民医保制度改革等建议提案有效促进了民生改善。免费义务教育全面实现，教育资助体系实现全覆盖，新建本科院校1所、高职学院6所，实现县县有高校。卫生计生事业长足发展，群众健康水平显著提高，全市人均预期寿命从2011年的74.86岁提高到79.94岁。平安德阳建设扎实推进，群众对社会治安满意率连创新高。圆满完成对口援建芦山和首轮援藏工作。

五年来取得的成绩，是在党中央国务院、省委省政府和市委的正确领导下，在市人大、市政协监督支持下，全市广大干部群众齐心协力、共同奋斗的结果。在此，我代表市人民政府，向市人大代表、政协委员和离退休老同志，向各民主党派、工商联、无党派人士、人民团体和全市人民，向驻地人民解放军、武警官兵和公安干警，向所有关心、支持德阳发展的海内外朋友，表示衷心的感谢，并致以崇高的敬意！

成绩鼓舞人心，差距必须正视。当前，全省市州竞相发展态势逼人，德阳被赶超压力空前加大；经济下行压力较大，部分指标与预期目标存在一定差距；创新驱动转型发展任重道远；一市一区格局制约发展，核心功能品质和承载能力亟待提升；做强县域经济、统筹城乡发展的任务较重；生态保护体系不够健全，环境质量需进一步提高；群众反映的一些突出问题尚未得到妥善解决，保障和改善民生任务艰巨；政府工作还存在不足，一些领域的事中事后监管不到位，少数政府工作人员面对新形势不适应、不作为、不会为、不善为的问题比较突出。对此，我们将高度重视，对标先进找差距，奋力赶超争一流，扎实做好转型发展、加快发展这篇大文章。

今后五年的奋斗目标和主要任务

市第八次党代会深刻分析了今后五年面临的机遇和挑战，明确了“建设成都国际化大都市北部新城”战略定位，我们要敢于担当，务实作为，奋力走出一条具有德阳特色的发展道路。

今后五年，政府工作的总体要求是：高举中国特色社会主义伟大旗帜，全面贯彻党的十八大和十八届三中、四中、五中、六中全会精神，以邓小平理论、“三个代表”重要思想、科学发展观为指导，深入学习贯彻习近平总书记系列重要讲话精神，按照“五位一体”总体布局和“四个全面”战略布局，积极践行“五大发展理念”，深入实施省委“三大发展战略”，全面落实市第八次党代会部署，坚持科学发展、加快发展工作主基调，保持专注发展、转型发展战略定力，抓实全面创新改革和成德同城化两个“一号工程”，聚力经济发展和脱贫攻坚两个主战场，筑牢平安德阳和民生保障两条底线，加快建成成都国际化大都市北部新城，加快迈入转型发展快车道，奋力实现“五个走在前列”，率先在全省全面建成小康社会。

今后五年，经济社会发展的奋斗目标是：按照“十三五”规划和市第八次党代会提出的目标任务，力争到2020年，地区生产总值突破2500亿元，全社会固定资产投资年均增长12%以上，一般公共预算收入年均增长10%以上，规上工业增加值年均增长9%以上，服务业增加值突破900亿元，社会消费品零售总额突破1200亿元，城乡居民人均可支配收入分别突破4万元、2万元，粮食总产稳定在185万吨以上，城镇化率达到55%以上，全面完成节能减排任务。

今后五年，政府工作的主要任务是：

——坚定不移落实全市重大战略部署，奋力开拓德阳发展新境界。战略问题是一个地区发展的根本性问题。市第八次党代会站在时代前沿和战略全局高度作出的战略部署、描绘的美好蓝图，是德阳发展的总纲领。我们必须深刻领会，统筹安排，协调推进，酌缓急而布之，连上下而通之，衡内外而施之，努力把宏伟蓝图变成美好现实。要以“建设成都国际化大都市北部新城”目标为引领，按照“12345”发展路径，强化“投资+创新”双轮驱动，大力振兴实体经济，加快发展民营经济，发展特色县域经济，优化经济结构，深化改革开放，在创新理念中赢得主动，在保持定力中赢得优势，在敢于担当中赢得未来，在务实重行中实现新一轮腾飞。

——坚定不移实施创新驱动发展战略，奋力推动经济迈上新台阶。当今时代，惟创新者进，惟创新者强，惟创新者胜。全面创新改革、大众创业万众创新、创新驱动转型发展，是德阳发展的当务之急。我们必须把准时代脉搏，抢抓战略机遇，闯过转型关，开创新局面。要举全市之力、集全市之智推进全面创新改革“一号工程”，全力争创国家创新型城市、国家制造业创新中心，加快建设装备技术智能制造示范市。要广泛开展大众创业万众创新，加大“十百千”创新型企业培育力度，支持企业家专心创新创业，促进新动能发展壮大、传统动能焕发生机，力争2018年地区生产总值突破2000亿元，经济实力迈上一个崭新台阶。

——坚定不移推进全域一体统筹发展，奋力谱写城乡建设新篇章。不谋全局者，不足谋一域。成德同城化、全域城镇化，既是区域一体化发展大趋势，也是德阳发展的重中之重。我们必须跳出单打独斗的小格局，以着眼全域的大视野，主动融入“大成都都市圈”，赢得更大发展空间。要牢固树立“大德阳”理念，以成德同城化带动全域城镇化，以全域城镇化推进城乡一体化。要开展新一轮交通建设大会战，3年建成天府大道北延线，加快构建“五环多轴”交通体系，全面对接成德轨道交通规划。要有力有序推进行政区划调整，打造“双百”现代化大城市，加快县域城市建设，深入实施“百镇建设行动”，建成幸福美丽新村1200个，组成一支整体实力强劲的“联合舰队”。

——坚定不移全面深化改革扩大开放，奋力增创跨越发展新优势。改革开放是发展进步的活力之源。全面深化改革、供给侧结构性改革、“放管服”改革、重点领域改革，是德阳改革发展的重任。我们必须坚持“两点论”找好平衡点，坚持“重点论”抓住关键点，促量变到质变，积小胜为大胜，趟过深水区，开辟新天地。要突出抓好供给侧结构性改革，落实“三去一降一补”“一提一创一培”。要以壮士断腕的勇气深化“放管服”改革，以“工匠精神”组织实施改革。要全面深化重点领域和关键环节改革，抓实农业农村、国资国企、投融资等改革创新。要实施充分开放合作战略，成功创建综合保税区，力争五年到位市外资金3500亿元，进入全省第一梯队。

——坚定不移守住底线增进民生福祉，奋力创造幸福美好新生活。底线是不可逾越的警戒线。经济增长的合理区间、全面小康的时间节点、金融债务的风险防范、安全稳定的社会局面、持续改善的生态环境，是德阳发展的底线。我们必须居安思危、未雨绸缪，曲突徙薪、管控风险，兜住民生底线，织牢安全防线，守住生态红线，在牢牢守底中稳稳托底，在稳稳托底中奋力夺取全面小康新胜利。要以更高标准推进脱贫攻坚，确保2017年率先全面脱贫。更大力度提升民生保障水平，抓好民生工程和民生实事。更高起点提高公共服务能力，把教育、卫生、文化等事业做优、产业做强。更大决心打好污染防治“三大战役”，让“蓝天常在、青山常在、绿水常在”成为德阳未来新常态。

——坚定不移建设法治政府法治德阳，奋力开创依法治理新局面。法者，国之重器。职能科学、权责法定、执法严明、公开公正、廉洁高效、守法诚信，是法治政府建设的目标。我们必须恪守“法”的尺度、遵循“法”的治理，自觉运用法治思维和法治方式推动工作，依法全面履行政府职能。要树立“法定职责必须为”的思想，把该管的事情管住管好。要绷紧“法无授权不可为”的弦，把该放的权力应放尽放。要完善权力运行制约和监督机制，推进依法决策、依法管理、依法监督。要以依法行政带动法治社会建设，让全民学法、守法、用法成为社会新风尚。

我们坚信，经过全市上下的奋发努力，新一届政府的奋斗目标一定会如期实现，一个经济更加发达、文化更加繁荣、生态更加优美、社会更加和谐、群众更加幸福的崭新德阳，必将展现在全市人民面前！

2017年工作安排

2017年是实施“十三五”规划的重要一年，是供给侧结构性改革的深化之年，也是新一届政府履职的第一年。做好今年工作，意义重大。我们要按照中央、省委、市委经济工作会议部署，坚持稳中求进工作总基调，坚持以提高质量和效益为中心，坚持以推进供给侧结构性改革为主线，在巩固“稳”的基础上下功夫，在积蓄“进”的力量上出实招，促进经济平稳健康发展和社会和谐稳定。

今年经济社会发展主要预期目标是：地区生产总值增长8.5%左右；全社会固定资产投资增长13%；一般公共预算收入同口径增长10%；规模以上工业增加值增长10%；服务业增加值增长10%；社会消费品零售总额增长12.5%左右；城乡居民人均可支配收入分别增长8.5%左右、9%以上；城镇登记失业率控制在4.2%以内；节能减排达到国家和省的要求。为此，要抓好以下六方面工作。

一、着力稳定经济运行，持续保持“双中高”发展态势

大力提升投资消费持续拉动力。巩固全市上下大抓项目的良好态势，继续开展“项目年”活动。抓好73个省重点项目和130个市重点项目建设。抓好项目前期工作，主动对接国家重点投资领域和国家专项建设基金投资方向，策划、包装、储备一批项目。抓好新签约项目履约率和资金到位率，重点推动首控德阳示范性科教城、中电国际天然气分布式能源等项目落地。抓好在建项目建设进度，强化分类调度和要素保障。通过PPP等模式，扩大有效投资。着力促进消费扩大和升级，推进传统消费向个性化、品质化转变，培育教育文化、健康养老、旅游休闲等更多消费热点。

大力提升先进制造业发展水平。振兴实体经济的主战场在制造业。深入实施“中国制造2025”，加快建设装备技术智能制造示范市，突出抓好“互联网+智能制造”“装备制造大数据”，优化提升“德阳装备制造工业云”，力争建成30个智能工厂和数字化车间。实施“传统产业振兴计划”，加快推进184个总投资2000万元以上重点工业及技改项目，打造千亿医药产业，建设新型化工基地、烟草基地。实施“高端新兴产业培育计划”，依托重型燃气轮机、铝空动力、石墨烯等重点项目，大力发展高端装备、新能源、新材料、节能环保等新兴产业，战略性新兴产业增加值增长16%以上。

大力提升现代服务业聚集辐射力。深化省级现代服务业综合改革试点，深入实施“服务业三百工程”，力争服务业增加值突破600亿元。推进中心城区服务业转型发展，培育壮大总部经济、楼宇经济、会展经济。加快发展“五大新兴先导型服务业”，促进中国微创国际医学中心、中国非物质文化遗产传承中心等一批重大项目尽快落地。推动实体零售业线上线下融合发展。推进现代服务业与先进制造业融合发展，培育和引进一批具有区域影响力的生产性服务企业。加快全域旅游发展，放大三星堆、三国文化吸引力，大力开发环龙门山、龙泉山旅游产业带，突出打造中国玫瑰谷、雪茄风情小镇、航空小镇、西海生态小镇等特色小镇，加快建设一批乡村康养休闲旅游基地，实现旅游收入230亿元。

大力提升现代农业发展质量效益。深入实施“三优两高”现代农业发展战略，着力打造全省现代农业先行示范区。深入推进农业供给侧结构性改革，把增加绿色优质农产品供给放在突出位置，狠抓农产品标准化生产、品牌创建、质量安全监管，促进农业提质增效。加快推进农产品精深加工业发展，抓好现代农业示范园区建设，打造一二三产业融合发展示范园区10个。提高粮食综合生产能力，打造现代粮食产业基地，全市粮食总产稳定在190万吨以上。大力发展观光农业、生态农业、都市农业、创意农业和现代畜牧业，广辟农民增收致富门路。做好第三次农业普查工作。

大力提升产业园区规模集聚效应。实施园区提档升级，全面提升产业承载能力和配套服务能力。狠抓重点项目形象进度，着力把德阳经开区和高新区打造成引领创新发展的千亿产业新城。打造国际领先的智能制造产业集群，重点发展清洁能源装备、节能环保装备、通用航空、医药健康等专业特色园区，支持绵竹经开区、什邡经开区创建省级高新区，支持成德工业园、德阿产业园创建省级开发区。打造区域性现代服务业聚集区，加快建设“中欧班列”德阳现代物流港，推动建立跨境电子商务示范园区。加大现代金融产业园招商力度，积极引入各类股权投资机构、信用评级公司等，推动金融机构集聚发展。

二、狠抓全创一号工程，打造创新驱动发展新引擎

坚定不移推进全面创新改革。加快建设国家高端装备产业创新发展示范基地，塑造德阳制造新名片、提升国际竞争新优势。抓好“9张清单”落实落地，总结提炼一批可复制可推广的典型经验。支持187项关键核心技术攻关，推进132项科技成果转化，培育一批重大创新成果，力争高新技术产业产值达到1200亿元。推动成德绵创建国家军民融合深度发展示范区，重点抓好德阳军民融合创新产业园、明日宇航国际航空零部件基地建设，帮助本地企业获取军品生产资质，军民融合产值增长15%以上。推进职业教育与产业发展深度融合，建立四川装备技术产教联盟，加快建设中德汽车学院、库卡机器人学院、西门子先进制造中心等项目。

多措并举深入推进创新创业。全方位发挥“双创”乘数效应，深化“互联网+”行动，积极创建“双创”示范基地。更好发挥企业创新主体作用，促进“四新经济”茁壮成长。实施更积极、更开放、更有效的人才引进政策，抓好“全面创新（国际）领军人才服务中心”“千人专家德阳创新创业孵化中心”建设，大力引进教育、卫生、科技等高层次人才，打造助推创新驱动发展、领先西部的人才集聚区。深入开展“德阳创客”品牌活动，举办创新创业大赛、“中德青年文创周”等系列活动，打造各具特色的“众创空间”。深入实施知识产权强市战略，大力推进国家专利质押融资及专利保险试点工作。

促进科技金融产业融合发展。全面深化产融结合，搭建科技金融合作平台，壮大天使投资引导基金规模，建立股权投资、风险补偿、专利质押运营等基金投入机制，为科技型企业提供全生命周期的金融服务。加强金融创新，支持商业银行成立科技信贷专营机构，推动国机集团发起设立金融租赁公司，支持发行战略性新兴产业专项债券等融资产品。优化财政投入模式，通过贷款贴息、投资基金、风险融资等方式，撬动社会资本投入，增强“首台套”保险实施精准度。扩大中小企业债务融资工具及规模，培育和支持60家企业挂牌上市。加强风险监测预警，切实防范化解金融风险。

三、深化成德同城发展，推动实现全域新型城镇化

打响新一轮交通建设大会战。成德同城，交通先行。着力抓好总投资1200亿元的42个交通重点项目，加快构建高效、安全、便捷、多层次的成德组合型交通体系。聚力建设以“五环多轴”为骨架的大城市框架，强力推进天府大道北延线、什德中、德罗、德绵干道建设，改造升级一环路，启动建设二环路，规划建设连接各县（市）的三环路，推进成都经济区环线高速公路德阳段建设，尽快启动成德绵高速扩容和旌江干线、成德大道、108国道提档升级，规划中心城区连接各县（市）的第二城市干道。新（改）建农村公路240公里。有序推进成德公交一体化建设，优化完善城市公交运行体系。对接成都轨道交通远景规划，制定市域轨道交通规划。

构建“四位一体”全域城镇体系。以成德同城化带动全域城镇化，全力抓好“六个协同”，推动形成“一核两中心”格局。按照全域市辖区的格局，有序推进区划调整，完成罗江县撤县设区，启动广汉市撤市设区，推动什邡市、绵竹市撤市设区和中江县行政区划调整。加大全域城市建设力度，围绕“南进北优、西扩东拓”总体布局，打造“双百”现代化大城市，全面提升卫星县城承载力，切实推进“城市升级三年行动”“三大改造”“四化工程”，实施道路交通、农贸市场、旌湖两岸、五大湖森林公园、南大门等一批提档升级项目，推进凯江桥西头下穿等交通堵点立交道路建设，建成地下综合管廊4.18公里。完成八角水库建设，加快华强沟、石泉水库建设。按照“七个一”标准推进重点镇建设，创建省级“四好”幸福美丽新村173个。

着力打造美丽德阳新家园。全力争创全国文明城市，大力提升市民文明素质。拓展绿色生活空间，突出打造“德阳国际健康谷”，加快构建“一谷两江五湖多园”的中心城区生态格局。坚决打赢污染防治“三大战役”，实施重点流域整治项目15个，建成城乡污水处理厂（站）65个，加大黑臭水体综合整治力度，全面强化大气、土壤污染防治。加强环境执法，完善联动机制，着力解决群众反映强烈的环境问题。开展大规模绿化行动，推行园林城市全域创建，确保顺利通过国家卫生城市、园林城市、森林城市复查，举办首届园艺博览会，有序推进大熊猫国家公园建设。全面完成节能减排财政政策综合示范市目标任务，抓好磷石膏综合利用，积极争取城市静脉产业基地国家试点。

四、加大改革开放力度，不断厚植转型发展新动能

深化供给侧结构性改革。扎实落实“1+5”方案，减少无效供给，扩大有效供给。强化引导去产能，推动煤矿整体退出，“一企一策”妥善处置“僵尸企业”。因城施策去库存，提高货币化安置比例，全市商品住房库存面积控制在280万平方米以内。分类有序去杠杆，支持企业利用资本市场直接融资，力争全年超过180亿元；加强财政管理，确保政府性债务安全可控。帮助企业降成本，实行收费目录清单管理，降低各类中介评估费用，扩大直购电直供气试点范围。狠抓“一提一创一培”，发扬“工匠精神”，培育“百年老店”，开展品牌创建和质量对标提升行动，争创全国质量强市示范城市，加强全国知名品牌示范区建设。

突出抓好重点领域改革。深化“放管服”改革，扎实开展相对集中行政许可权改革试点，全面推行“双随机、一公开”，推进交通、文化、旅游、城管四大领域综合行政执法体制改革试点，推动执法重心下移全覆盖。深化商事制度改革，激发民营经济发展活力。深入推进市属国有停产企业“人资分离”改革试点，加快退出一般竞争性领域。推进农村土地“三权”分置改革，加快建设成德农村产权交易平台，全面建成市、县、乡三级农村产权流转交易平台。推进县级农村信用联社改制组建农商行，力争德阳农商行挂牌。

实施充分开放合作战略。积极融入“一带一路”倡议，落实“251三年行动计划”，打造“一带一路”重大装备供应商。围绕高端发展“招大引强”、转型升级“补链强链”，推进专业招商、精准招商，力争到位市外资金700亿元。举办首届“四川国际航空航天展”，积极参加“中外知名企业四川行”“万企出国门”等活动。加强与长三角、珠三角等国内重点地区的经济合作，积极承接产业转移。推动对外贸易便利化，深化“大通关建设”，加快筹建综合保税区，争取共享自贸区政策，力促进出口企稳回升。

五、奋力冲刺全面小康，提升人民群众的幸福指数

率先全面完成脱贫攻坚。大力推进产业扶贫、新村建设扶贫、贫困家庭技能培训等12个专项扶贫工作，深化“三免三定”、股权量化等特色扶贫，确保3.85万贫困人口全面脱贫，市级贫困村全部退出。按照贫困户“一超六有”、贫困村“一低五有”退出标准，抓好集中退出验收考核和第三方检查评估工作，确保脱真贫、真脱贫。扎实推进对口帮扶藏区彝区工作，确保6个受扶县贫困村、贫困户如期脱贫。

切实提高民生保障水平。深入实施民生工程，办好民生实事。实施大学生创业引领计划，统筹抓好高校毕业生、农村转移劳动力等重点人群的就业工作，新增就业3.3万人。加强“五险统征”，巩固城乡居民医保制度，健全重大疾病救助体系，完善城乡居民大病保险制度和城镇职工补充医疗保险制度。健全社会养老体系，建设日间照料中心89个。实施棚改8023户，完成农村危房改造7966户。

切实增强公共服务能力。大力发展优质教育，助推城市品质跃升。新建10所幼儿园、3所小学，改善30所农村义务教育薄弱学校基本办学条件。着力打造职业教育促进经济社会发展示范市。加快建设电子科大成都学院什邡校区、国家法官学院四川分院、标榜职业学院中江校区等项目。深化医药卫生体制改革，建立“三医”联动机制，以家庭医生签约服务为抓手助推分级诊疗，推进健康城市及医养结合试点建设，提升重大疾病防治能力，开展计生特别扶助对象“三项保险”试点。深入开展“三讲三爱两进步”活动，建设省级文创产业示范园。举办全市第三届运动会，抓好全民健身苑建设。

切实推进社会治理创新。深化平安德阳建设，深入实施“主动警务战略”，完善立体化治安防控体系，构建“大平安”社会管理格局。健全重大决策社会稳定风险评估机制，坚持用法治思维化解信访矛盾。推进村（社区）综合服务信息平台建设，深化社区网格化管理。完善安全监管机制，完成“智慧安监”大平台建设，坚决遏制重特大安全事故发生。健全覆盖城乡的食品药品安全监管体系。强化国防动员和后备力量建设。推进防灾减灾救灾体制机制改革，提高突发事件应急处置能力，确保城乡安全、市民安宁、社会安定。

六、加强政府自身建设，切实树立为民务实新形象

建设法治政府。自觉接受市人大及其常委会的法律监督和工作监督，主动接受市政协的民主监督，虚心接受社会监督和舆论监督。切实做好建议提案办理工作，广泛听取民主党派、工商联、无党派人士和人民团体的意见。充分发挥政府在立法工作中的依托作用。健全依法决策、科学决策、民主决策机制，严格落实重大行政决策法定程序。坚持依法行政，加强和改进执法监督。全面推进“七五”普法，深入开展“法律七进”。完善公共法律服务体系，加强法律援助工作。

建设服务型政府。优化审批服务，完善“一诺即办”“一窗进出”“全程代办”，促进审批提速增效“零超时”。推行“互联网+政务服务”，完善“政务云”，提升网上办事大厅功能，拓展微信“城市服务”，让数据“多走路”、群众“少跑腿”。强化政府信息公开和新闻发布，做好行政权力依法规范公开运行工作，推进公共权力、公共资源、公共服务公开透明。完善“12345”政府服务直通车运行机制。拓展政府购买公共服务范围，提高公共服务质量和效率。强化工作落实，提高政府执行力。

建设廉洁政府。认真贯彻全面从严治党要求，持续深化巩固良好政治生态。严格落实党风廉政建设责任制，健全重点领域廉政风险防控制度，完善惩治和预防腐败体系，实现干部清正、政府清廉、政治清明。推行容错纠错、澄清保护机制，健全正向激励机制，促进干部创造性开展工作。全体公务员要牢记历史使命，在不忘初心中行稳致远，在不忘本来中开辟未来，永葆奋斗精神，坚持克难奋进，努力交出无愧于时代、无愧于人民的崭新答卷。

各位代表！德阳已经站在一个新的发展起点上，具备了更加坚实的发展基础，积蓄了更加充足的发展动力。让我们紧密团结在以习近平同志为核心的党中央周围，在省委、省政府和市委的坚强领导下，坚定信心，务实重行，开拓奋进，为建设成都国际化大都市北部新城，率先在全省全面建成小康社会而努力奋斗，以优异成绩迎接党的十九大胜利召开！
